# Engrailed 2 (EN2) acts as a glioma suppressor by inhibiting tumor proliferation/invasion and enhancing sensitivity to temozolomide

**DOI:** 10.1186/s12935-020-1145-y

**Published:** 2020-03-02

**Authors:** Tengfei Li, Wanchun Yang, Mao Li, Shuxin Zhang, Xingwang Zhou, Mingrong Zuo, Qiuyun Yuan, Mina Chen, Yanhui Liu

**Affiliations:** 1 Department of Neurosurgery, State Key Laboratory of Biotherapy, West China Hospital, Sichuan University, 37 Guoxue Alley, Chengdu, 610041 Sichuan People’s Republic of China; 2 Neuroscience & Metabolism Research, State Key Laboratory of Biotherapy, West China Hospital, Sichuan University, 37 Guoxue Alley, Chengdu, 610041 Sichuan People’s Republic of China

**Keywords:** EN2, Glioma, Temozolomide, Cell invasion

## Abstract

**Background:**

Glioma is one of the most malignant brain tumors and accounts for the majority of brain cancer related death. Despite progress on mechanistic studies, current understandings of the initiation and progression of glioma are still incomplete. Previous studies demonstrate that *Engrailed*-*2* (EN2), a homeobox-containing transcription factor, is associated with tumorigenesis in a range of cancers heterogeneously, however, the profiles of EN2 expression and its potential functions in gliomas remain unclear.

**Methods:**

Real-time PCR was used to identify the expression of EN2 in glioma tissues. To study the biological function of EN2 in glioma, we compared the cell viability and proliferation profiles between EN2 overexpressed and control cells using cell counting kit-8 (CCK8) assay, EdU incorporation assay and colony formation assay. Flow cytometry and Hoechst staining assays were performed to investigate the role of EN2 on glioma cell death. Finally, wound healing and transwell assays were carried out to investigate the role of EN2 on glioma cell invasion.

**Results:**

We identified that EN2 was downregulated in human gliomas compared with paired adjacent normal tissues and negatively associated with glioma malignancy. Elevated EN2 expression inhibits cell proliferation, enhances glioma sensitivity to temozolomide and inhibits migration/invasion of glioma cells.

**Conclusions:**

Our data identify a novel function of EN2 in glioma suppression and provide potential therapeutic targets for glioma therapy.

## Background

Glioma is one of the most prevalent malignant and lethal tumors in the central nervous system, which displays aggressive growth and diffuse invasion [1, 2]. Gliomas including astrocytomas, oligodendrogliomas and glioblastoma multiforms (GBM), are classified as grade I to IV based on histological features and genetic alterations [3]. Despite standard treatment comprised of maximal safe surgical resection, radiotherapy plus concomitant and maintenance temozolomide chemotherapy [4, 5], the prognosis for glioma patients remains poor and almost all patients relapse inevitably. Particularly, the median survival time of patients with GBM remains approximately 1 year, and the 5-year overall survival rate is less than 5% [6, 7].

Glioma evolves rapidly by acquiring new mutations or altered gene expressions for drug resistance [8]. Studies have interrogated a network of gene expression profiles to identify novel genes and critical pathways for glioma malignancy [9]. Transcription factor (TF) regulates gene expression by translating *cis*-regulatory codes into specific gene-regulatory events [10], and acts as a functional unit in cell fate decisions in many cell types and systems including cancer. Therefore, electing TF as a rational target for cancer therapy is a promising concept [11]. Even though progressive technologies and systematic analysis have cataloged a landscape of TF alternations and gene expression changes in glioma [12–15], current understandings of gliomagenesis are still incomplete.

*Engrailed*-*2* (EN2) gene encodes a homeobox-containing transcription factor and participates in the regionalization, patterning and cell differentiation in early brain development [15–17]. Accumulated evidence demonstrates that EN2 is highly associated with tumorigenesis in some types of cancers [18–26], however, controversial results show that EN2 is silenced in other cancers [27, 28]. As for glioma, it remains unclear about EN2 expression pattern and potential functions. Here, using clinical samples combined with functional approaches, we demonstrate that EN2 is a novel suppressor of glioma tumorigenesis. EN2 expression is downregulated in human gliomas compared to adjacent brain tissues, which is negatively associated with glioma malignancy. Elevated EN2 expression inhibits cell proliferation and enhances glioma sensitivity to temozolomide. Moreover, EN2 blocks the invasion of glioma cells by inhibiting MMP9 expression. Our data identify a novel function of EN2 in glioma suppression and provides potential targets for glioma therapy.

## Materials and methods

### Patients and samples

**Fig. 1:**
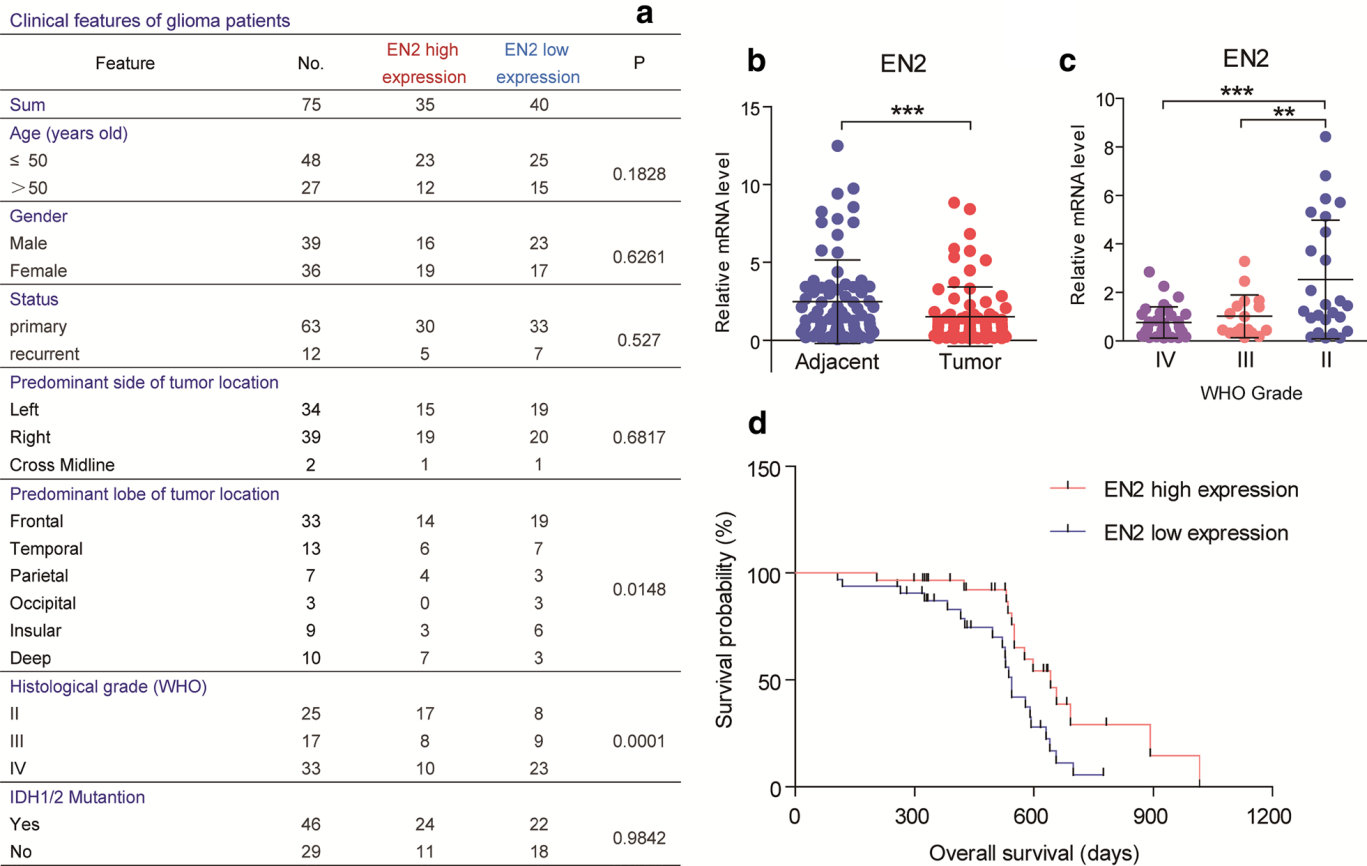
EN2 expression is negatively associated with glioma malignancy. **a** Stratified EN2 expression profiles of 75 glioma patients showing that EN2 was not correlated to the age, gender, status of onset, predominant location in side, and IDH1/2 mutation, but associated with predominant lobe and histological grade significantly. **b** Real-time PCR results showing that EN2 mRNA was decreased in gliomas compared to adjacent brain tissues (n = 75). **c** EN2 mRNA was decreased in high-grade gliomas (WHO III and IV, n = 17 and 33) in contrast to low-grade gliomas (WHO II, n = 25). **d** Kaplan–Meier survival analysis revealing that patients with higher EN2 expression carried a significantly better prognosis than the lower (n = 75). **p < 0.01 and ***p < 0.001

All the human studies were approved by the Institutional Review Board of West China Hospital of Sichuan University and all patients provided written informed consent. A total of 75 patients, operated between Jun 2016 and Jan 2019 at the West China Hospital of Sichuan University with primary gliomas diagnosed, were included in this study to examine the mRNA levels of EN2 (Fig. 1a). The pathological diagnosis of glioma was confirmed and classified according to the World Health Organization (WHO) criteria by two clinical pathologists in a blinded manner.

### Cell culture

Human glioma cell line U251 was used to investigate EN2 function in this study. U251 cell line was purchased from the Shanghai Institute of Biochemistry and Cell Biology, Chinese academy of sciences (China). Cells were cultured in Dulbecco’s Modified Eagle’s Medium (DMEM, HyClone, Thermo Fisher Scientific, USA) supplemented with 10% fetal bovine serum (FBS, PAN Biotech, Germany) and 1% penicillin–streptomycin antibiotic mixture (Cellgro, USA). All cells were cultivated in an incubator with constant temperature containing 5% CO_2_ atmosphere at 37 °C.

### Lentiviral construct and overexpression

The EN2 lentiviral vector (Ubi-MCS-3FLAG-CBh-gcGFP-IRES-puromycin) and control lentiviral were constructed by Shanghai Genechem Co., Ltd. (China). For lentivirus transduction, U251 cells were cultured for 24 h in a 12-well plate, and transfection efficiency was determined by an inverted fluorescence microscope. Afterward, puromycin (2.0 µg/mL) was used to screen infected cells to acquire stable EN2-overexpressed cells (Flag-EN2) and control cells (Ctrl). Cells were harvested and validated for subsequent experiments.

### Transcriptome sequencing

Total RNA was isolated cell samples with Trizol reagent according to the manufacturer’s protocol, and sequenced by Novogene (China). Reads were mapped to GRCh37.p13 genome and annotation with STAR using ENCODE recommended arguments. Gene-level read counts generated by STAR were used for differential expression analysis with the DESeq2 package. Log2 fold change and adjusted p values of genes generated by DESeq2 were used for visualization using R.

### Cell viability and proliferation assay

Cell viability was determined using Cell Counting Kit-8 (Dojindo China, China). Briefly, U251 cells were seeded at a volume of 100 μL and density of 1500 cells/well counting by the hemocytometer in 96-well plates and incubated in 3 days for CCK8 assay every 24 h. CCK-8 solution (10 μL) at a 1:10 dilution with FBS-free DMEM (100 μL) was added to each well followed by a further 3 h incubation under 5% CO_2_ at 37 °C. Relative absorbance was automatically measured at 450 nm wavelength on a microplate reader. The cell viability was calculated by the mean optical density (OD, absorbance). All experiments were performed three times, and values were plotted by averaging duplicated wells.

The proliferation of U251 cells was detected by EdU incorporation assay according to the manufacturer’s protocol from the Cell-Light EdU (5-ethynyl-2′-deoxyuridine) Apollo-567 In Vitro Imaging Kit (RiboBio, China).

For the colony formation assay, U251 cells were suspended and plated in 10 cm plates at low density (200 cells/plate) and cultured in 10% FBS complete medium for 2 weeks. The cells were washed twice with phosphate-buffered saline and stained with 0.5% crystal violet for 20 min and photographed. The colony with more than 50 cells were considered for enumeration using an inverted microscope.

### Cell death assay

Early apoptosis of U251 cells was detected by Annexin V Alexa Fluor647/7-AAD kit (Beijing 4A Biotech, China) followed by flow cytometry on a Beckman cytoflex. Briefly, cells were plated in 6-wells plate overnight. When it was about 80–90% confluence, cells were digested, washed in pre-cooling PBS, then suspended in prepared binding buffer. 100 μL of suspension was transferred to a new tube followed by the addition of 10 μL of Annexin V Alexa Fluor647 and 5 μL of 7-AAD, then incubated in the dark at room temperature for 15 min and served it on ice followed by flow cytometry.

To induce cell death, U251 cells were treated with rotenone or temozolomide respectively. Rotenone (Sigma-Aldrich, USA) was dissolved in DMSO (Sigma-Aldrich, USA) with the final concentration of 1.0 μM. Temozolomide (Selleck Chemicals, China) was dissolved in DMSO with a final concentration of 100 μM. After incubation for 24 h, cells in 12-well cell culture plate with glass coverslips were stained by Hoechst 33258 (Beyotime, China) for staining and cell counting. The number of the apoptotic hyperchromatic nucleus and total nucleus were counted in five microscopic fields. The apoptosis index was defined as the mean ratio of apoptotic/total nuclear numbers.

### Wound healing assay

U251 cells were seeded in 6-well plates and incubated to reach 90% confluence. After serum-starved for 4 h, monolayers were scratched with a sterile 200-µL pipette tip to create a wound, and cells were then washed twice with FBS to remove floating cells. Subsequently, cells were cultured in serum-free DMEM medium for additional 48 h. Images were captured using an inverted phase contrast microscope at 0, 24 and 48 h, and analyzed using the wound-healing macro of ImageJ. The rate of wound closure was calculated based on the area of the gap.

### Cell migration and invasion assay

Chambers with 8.0 μm PET membranes in 24-well plates (Corning, USA) were used to measure cell invasion and migration. For migration assay, cells with a density of 2 × 10^4^ cells/well were seeded into the upper chamber of the 24-well transwell insert. The lower chamber was then filled with medium containing 10% FBS as a chemoattractant. The same number of cells were seeded in another well as a control. After 24 h, the upper surface of the membrane was scrubbed to remove all non-invaded cells. Cells migrated to the lower surface of the filter were fixed and stained with 0.5% crystal violet solution for counting and quantification. For invasion assay, cells were seeded into the upper chamber precoated with BioCoat Matrigel (BD, USA) according to the manufacturer’s protocol. After 48 h, the invasive ability was assessed the same as the migration assay. The invasion index was calculated by ImageJ. All assays were independently repeated for at least three times.

### Western blot

Total cell lysates were prepared by disrupting cells with 2% SDS lysis containing protease and phosphatase inhibitor cocktails (Thermo Scientific, USA). Protein concentrations were determined using the BCA Protein Assay Reagent (Thermo Scientific), then calibrated using a loading buffer. Equivalent proteins were loaded and separated by SDS-PAGE according to standard procedure. The primary antibodies used in this study were list below (Table 1).

**Table 1:** Antibodies used in this study

Antibodies		
Rabbit monoclonal EN2 antibody	Abcam	Cat#ab28731
Goat monoclonal EN2 antibody	Abcam	Cat#ab45867
Mouse monoclonal FLAG antibody	Sigma-Aldrich	Cat#F3165
Mouse monoclonal Beta actin antibody	Boster Biological Technology	Cat#BM0627
Rabbit monoclonal Vimentin antibody	Cell Signaling Technology	Cat#5741
Rabbit monoclonal E-Cadherin antibody	Cell Signaling Technology	Cat#3195
Rabbit polyclonal STAT3 antibody	Abclonal	Cat#A16975
Rabbit polyclonal MMP-9 antibody	Cell Signaling Technology	Cat#3852
Mouse monoclonal GAPDH antibody	Millipore	Cat#MAB374

### Immunofluorescence

For immunostaining, U251 cells were seeded in 12-well plates with coverslips (10^5^ cell/well) overnight, and fixed in 4% paraformaldehyde. After permeabilizing with 0.25% Triton X-100 for 10 min, cells were blocked in 10% goat serum and incubated with primary antibodies (anti-flag, 1:500) and secondary Alexa Fluor™ 594 Goat Anti-Mouse IgG (H+L) antibody (Invitrogen, USA). Slides were mounted using Prolong Gold antifade with DAPI. Images were acquired using a fluorescent microscope (Olympus, USA).

### RNA extraction and real-time PCR

For RNA extraction, glioma tissues or cells were homogenized, and total RNA was isolated using Trizol reagent (Invitrogen, USA) according to the manufacturer’s instruction. Reverse transcriptase reactions were performed by incubating 400 ng RNA with the first-strand cDNA synthesis kit for subsequent real-time PCR assay. The 2^−ΔΔCq^ method was used to calculate gene transcription level, with β-actin mRNA as control. Primer sequences were as list below (Table 2).

**Table 2:** Real-time PCR primers

Targets	Forward 5′-3′	Reverse 5′-3′
EN2	CCGGCGTGGGTCTACTGTA	CCTCTTTGTTCGGGTTCTTCTT
MMP1	GGGGCTTTGATGTACCCTAGC	TGTCACACGCTTTTGGGGTTT
MMP3	CGGTTCCGCCTGTCTCAAG	CGCCAAAAGTGCCTGTCTT
MMP9	GGGACGCAGACATCGTCATC	TCGTCATCGTCGAAATGGGC
β-actin	CATGTACGTTGCTATCCAGGC	CTCCTTAATGTCACGCACGAT

### Statistical analysis

All the statistical analyses in this study were calculated by GraphPad Prism 7.0 software (GraphPad, USA). The measurement data were presented as the mean ± standard deviation (SD). Statistical comparison between two groups was performed by Student’s *t*-test, and one-way ANOVA followed by the Bonferroni multiple comparison post hoc tests for multi-group comparisons. Kaplan–Meier survival curve comparison was performed by the log-rank test. The sample sizes (n) were indicated in the figure legends. A p-value < 0.05 was considered statistically significant.

## Results

### EN2 expression is negatively associated with glioma malignancy

As a first step to identify the possible contributions of EN2 in gliomagenesis, we analyzed the EN2 gene expression profiles in tumor tissues compared with adjacent brain tissues in glioma patients from West China Hospital of Sichuan University (Fig. 1a). By real-time PCR assay, we found that the mRNA level of EN2 was decreased in gliomas compared to adjacent brain tissues (Fig. 1b). Further examinations confirmed that EN2 expression was decreased in high-grade gliomas (WHO III and IV) in contrast to low-grade gliomas (WHO II) (Fig. 1c), suggesting that EN2 expression is associated with lower glioma grade. We also examined the protein level of EN2 using currently commercial antibodies. However, these antibodies were not reliable to detect endogenous EN2 protein, because they cannot even recognize overexpressed EN2 in glioma cell lines (data not shown).

To investigate the correlation of EN2 expression with clinical significance in gliomas, we performed Kaplan–Meier survival analysis and found that gliomas with higher EN2 expression carried a significantly better prognosis than those with lower EN2 expression (Fig. 1d). Taken together, all these findings indicate that EN2 expression is associated with a reduced glioma malignancy.

### EN2 overexpression alters gene expression profiles in glioma cells

**Fig. 2:**
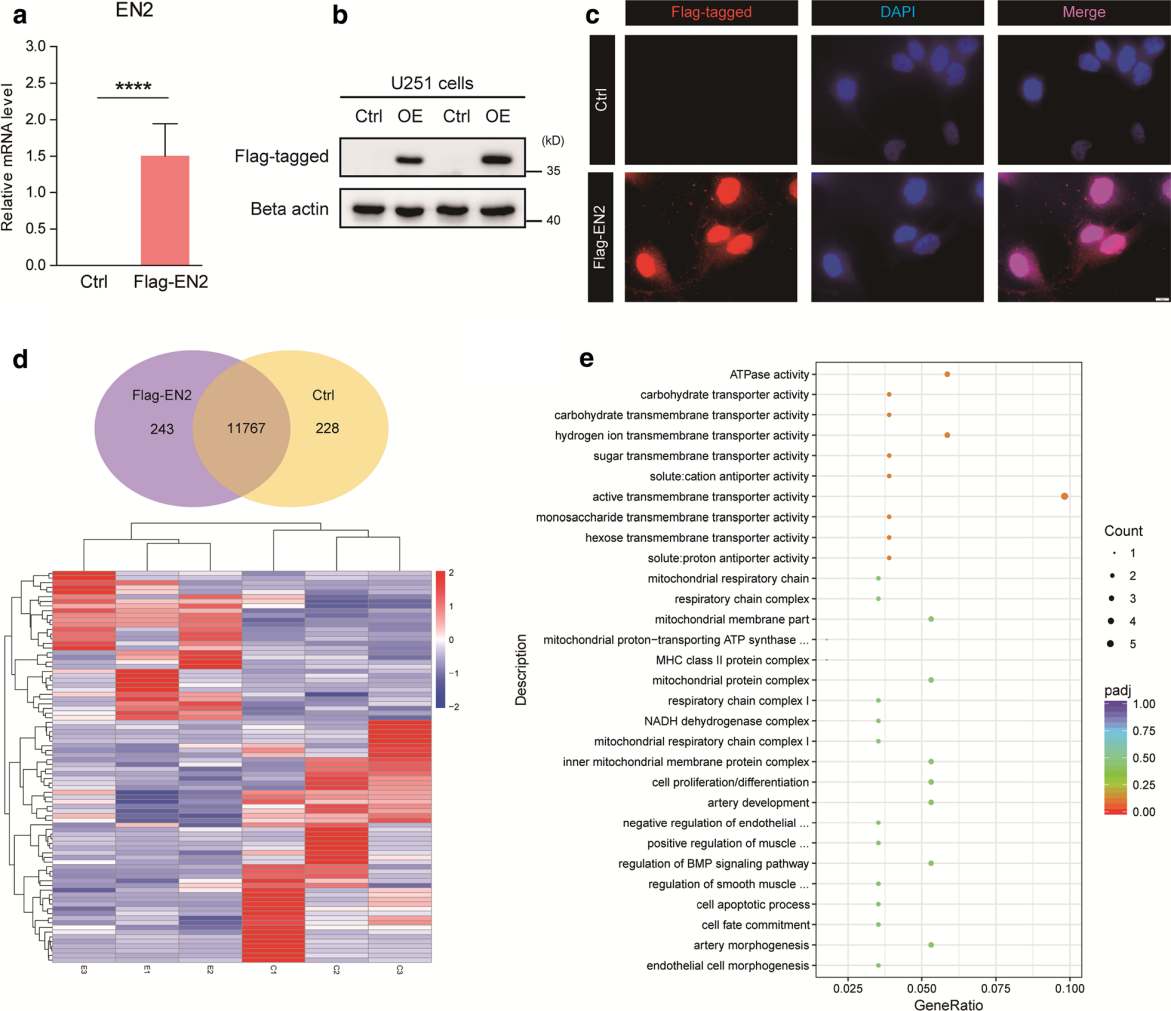
EN2 overexpression alters gene expression profiles in glioma cells. **a** and **b** Validation of EN2 overexpression in U251 cells by Real-time PCR and Western blot (n = 3). **c** Representative images indicating EN2 overexpression in U251 cells locating in the nucleus. **d** and **e** Altered gene profiles of EN2 overexpression by RNA-seq analysis (Ctrl, n = 3; OE, n = 3)

Since the expression of EN2 is inversely correlated with glioma malignancy, we sought to determine whether EN2 functionally suppresses gliomagenesis. We conducted a Flag-tagged EN2 overexpressed U251 human glioma cells, in which EN2 is lowly expressed. Real-time PCR and Western blot analysis validated that EN2 is successfully overexpressed in U251 cells (Fig. 2a, b). Moreover, immunostaining images confirmed that overexpressed EN2 was enriched in the nuclear, supporting the fact that EN2 is a homeobox-containing transcription factor (Fig. 2c). It’s also noted that EN2 signals were still existed in the cytoplasm, suggesting that EN2 may have functional roles beyond transcription factor.

To investigate the biological functions of EN2 in glioma cells, we performed transcriptome sequencing (RNA-seq) in Flag-EN2 overexpressed cells with matched controls. Results showed that Flag-EN2 altered 243 gene expression compared to controls (Fig. 2d). We found that EN2 overexpression regulated genes in cellular metabolism and transmembrane transport pathway curated by the Kyoto Encyclopedia of Genes and Genomes (KEGG) [29]. We also noted that the genes involved in mitochondrial oxidative phosphorylation (OXPHOs) and respiration machinery were controlled by EN2 (Fig. 2e). EN2 was reported as a transcription factor and therefore may serve as a regulator in nuclear-controlled mitochondrial metabolism. Nevertheless, considering that tumor cells are relying on accelerated nutrient transport and metabolism, it’s assumed that EN2 may control cell growth and survival in glioma cells. Indeed, we found that genes in cell proliferation/apoptosis were also controlled by EN2 (Fig. 2e), suggesting that EN2 may participate in glioma tumorigenesis.

### EN2 inhibits cell proliferation and promotes cell apoptosis in glioma cells

**Fig. 3:**
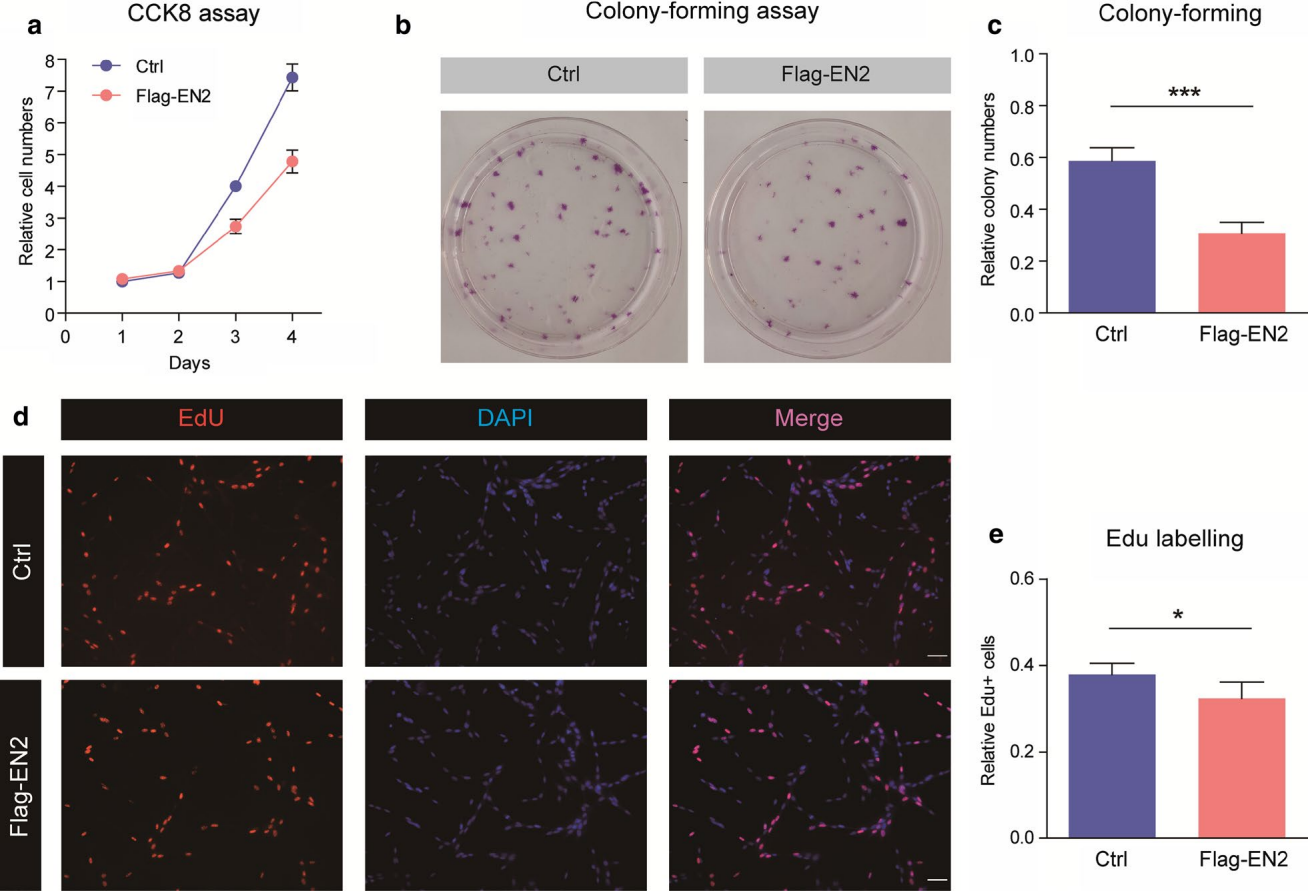
EN2 inhibits cell proliferation in glioma cells. **a** CCK-8 assay indicates that EN2 overexpression reduces cell proliferation in glioma cells (n = 3). **b** and **c** EN2 overexpression impairs colony formation in U251 cells (n = 4). **d** and **e** EdU incorporation assay show EN2 overexpression reduces EdU + cells in U251 cells (n = 6)

To investigate whether EN2 regulates glioma tumorigenesis, we evaluate cell proliferation/apoptosis in U251 cells transfected by Flag-EN2. Results from the CCK-8 assay indicated that EN2 overexpression reduced cell proliferation in glioma cells (Fig. 3a). Consistently, we performed colony formation assay and found that EN2 overexpression impaired colony formation in U251 cells (Fig. 3b, c). Moreover, we confirmed the effect of EN2 on cell proliferation by EdU labeling assay and found that EdU positive cells were slightly decreased in EN2 overexpressed cells, suggesting that EN2 exert an inhibitory effect on glioma proliferation.

**Fig. 4:**
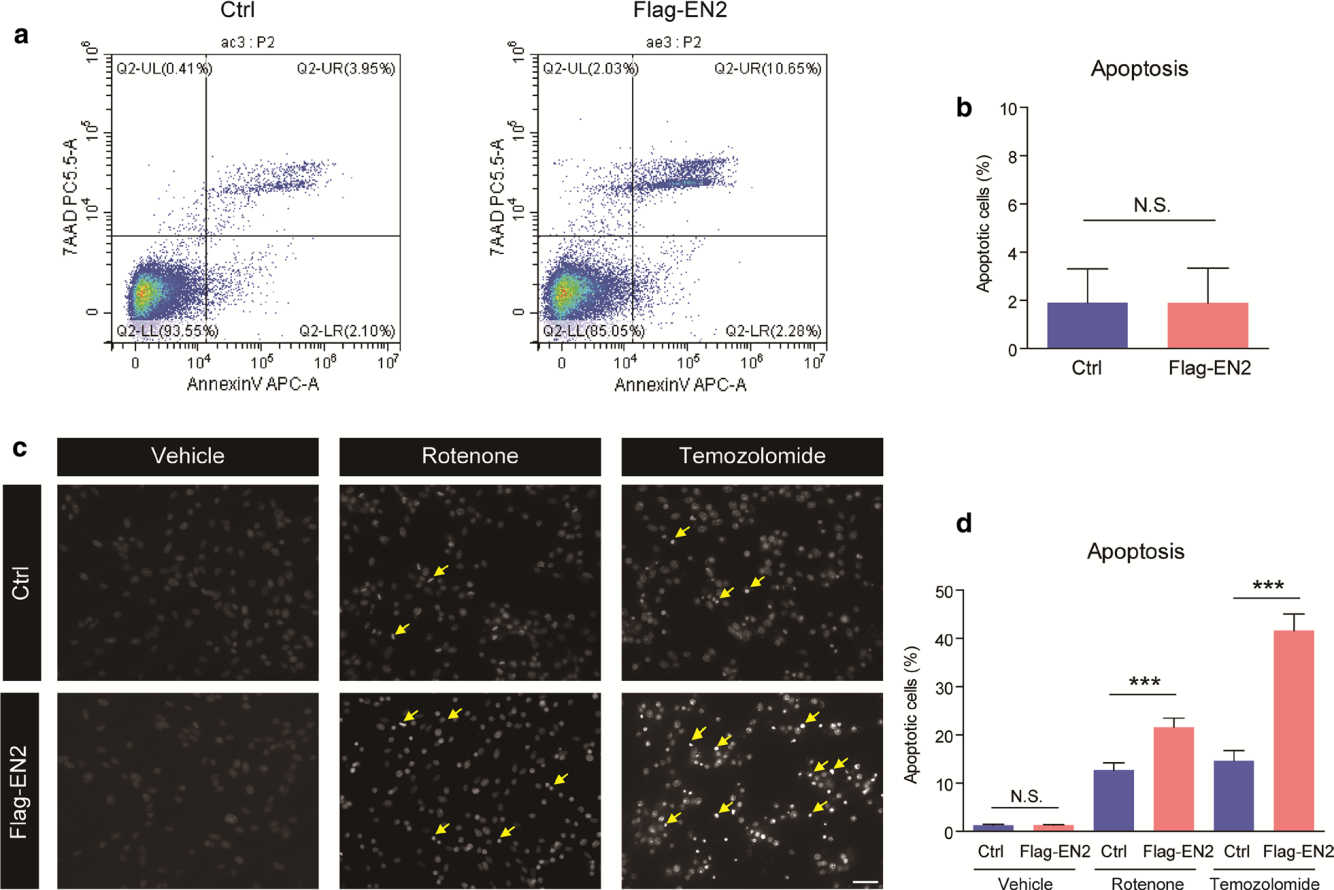
EN2 sensitizes glioma cells to temozolomide. **a** and **b** EN2 overexpression alone does not dramatically induce early apoptosis in U251 cells (n = 8). **c** and **d** EN2 overexpression promotes cell apoptosis after treatment of rotenone or temozolomide (n = 6). Scale bar, 20 μm

Next, we examined the effect of EN2 on cell survival/apoptosis in glioma cells. Results from flow-cytometry revealed that EN2 overexpression alone did not dramatically induce early apoptosis in U251 cells (Fig. 4a, b). However, by applying rotenone and temozolomide to induce apoptosis, it’s found that EN2 overexpression dramatically increased cell apoptosis under pharmacological treatment (Fig. 4c, d), indicating that EN2 sensitizes glioma cells to cell death and synergizes with temozolomide.

### EN2 suppresses cell migration/invasion in glioma cells

**Fig. 5:**
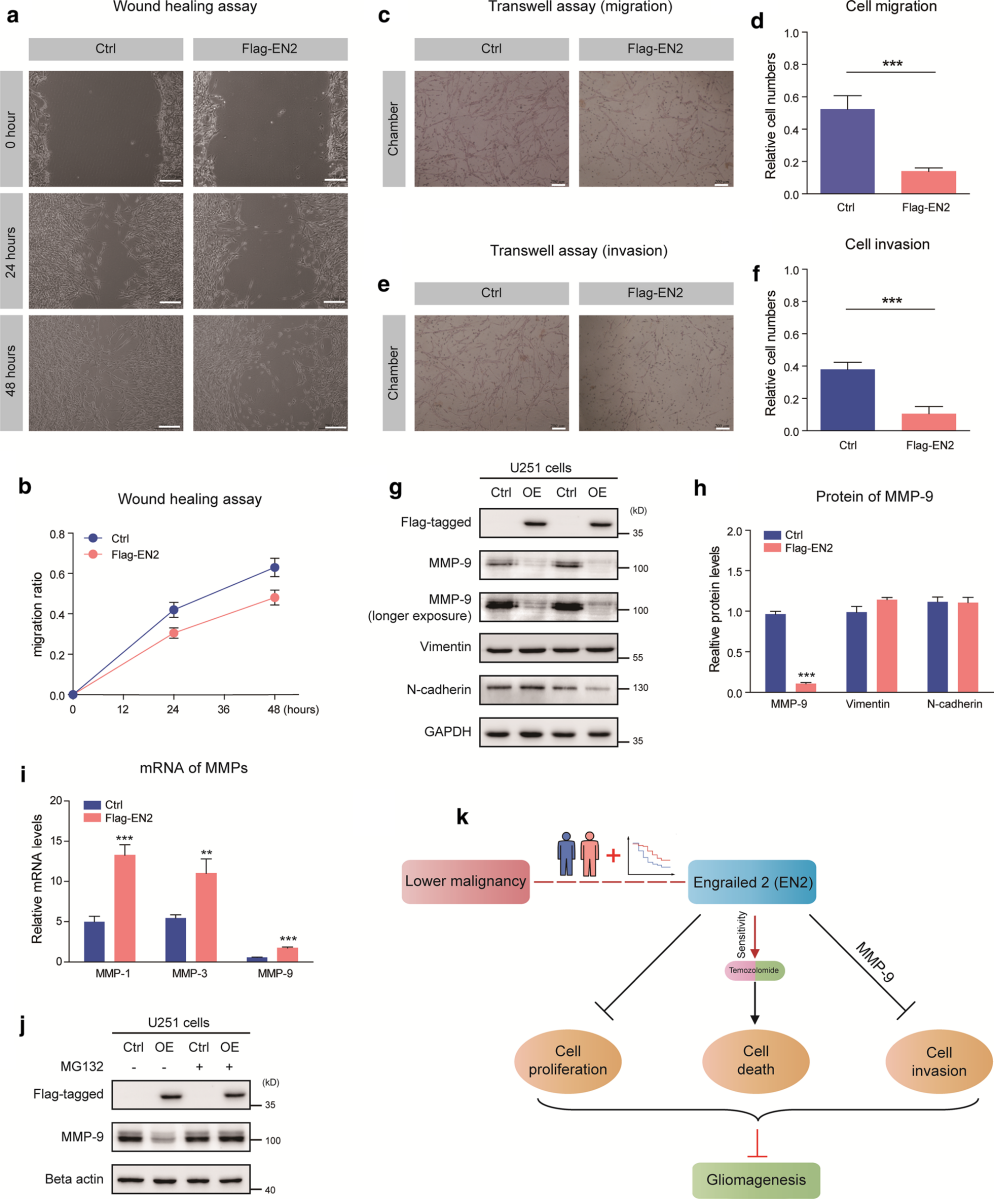
EN2 suppresses cell migration/invasion in glioma cells. **a** and **b** Wound healing assays demonstrate that EN2 overexpression inhibits the migration of U251 cells (n = 4). The migration of cells into the wound was assessed at 0, 24 h and 48 h. Scale bar, 200 μm. **c** and **d** EN2 overexpression reduces the rate of migration of U251 cells by Transwell migration assays (n = 4). Scale bar, 200 μm**. e** and **f** EN2 overexpression decreases the invasive ability of U251 cells by Transwell invasion assays (n = 5). Scale bar, 200 μm. **g** and **h** The level of MMP-9 protein are downregulated by EN2 overexpression. **i** MMP-9 mRNA is upregulated by EN2 overexpression (n = 3). **j** MG132 rescues MMP-9 proteins in EN2 overexpressed cells. **k** A schematic demonstrates that EN2 regulates cell proliferation/apoptosis/invasion of glioma cells

A typical feature of glioma is diffuse tumor invasion, and we sought to determine whether EN2 functionally suppresses glioma migration/invasion. Indeed, EN2 overexpression dramatically decreased the migration of U251 cells by wound healing assay (Fig. 5a, b) and transwell migration assay (Fig. 5c, d). Next, we evaluated the effect of EN2 on cell invasion by the Matrigel invasion assay. After 48 h of incubation, the numbers of cells invaded through the matrigel were calculated, as a result, EN2 overexpression strongly inhibited the invasive ability of U251 cells (Fig. 5e, f).

Tumor invasion is tightly controlled by multiple signaling pathways, including MMPs family. To reveal the molecular mechanism by which EN2 suppresses glioma cell migration/invasion, we examined and found that the protein level of MMP-9 was dramatically decreased by EN2 overexpression (Fig. 5g, h). However, it’s noticed that the mRNA level of MMP-9 was inversely increased by EN2 overexpression (Fig. 5i). Thus, we assume that EN2 may control MMP-9 protein stability/degradation in U251 cells. Indeed, the protein level of MMP-9 was restored by MG132, a proteasome inhibitor to block ubiquitin-conjugated protein degradation (Fig. 5j). Altogether, our results demonstrate that EN2 suppresses cell migration/invasion in glioma cells via MMP-9.

## Discussion

In this study, we investigate the potential role of EN2 in human gliomas, and reveal a novel function of EN2 in regulating cell proliferation/apoptosis/invasion and participating in gliomagenesis. EN2 is a suppressor of glioma tumorigenesis, which is downregulated in human gliomas and negatively associated with glioma malignancy. Elevated EN2 expression inhibits cell proliferation, enhances glioma sensitivity to temozolomide and blocks cell invasion of glioma cells. All these results imply a novel function of EN2 in glioma suppression and provide potential targets for glioma therapy (Fig. 5k).

EN2 is a member of the homeobox family, which encodes a homeodomain-containing transcription factor that is essential for early development. Elevation of EN2 has been highly correlated with the proliferation, invasion, and migration in several types of malignancies [18–26], however, controversial results show that EN2 is silenced in other cancers [27–29], and thus EN2 acts as a potential diagnostic and prognostic biomarker [23, 30–33]. However, decreased EN2 in renal carcinoma has been observed and downregulation of EN2 in 786-O cell line promoted proliferation and reduced apoptosis [27, 34, 35]. Thus, it’s proposed that EN2 has distinct functions in different types of tumors. In this study, we identify that EN2 is reciprocally associated with glioma malignancy. Low expression of EN2 is observed in the subtype of glioma patients with poor survival, suggesting that EN2 expression is a conceivable biomarker for glioma prognosis. Mechanistically, EN2 inhibits glioma proliferation and invasion, and induces apoptosis. Therefore, EN2 is a nodal point for precise regulation of gliomagenesis.

Mechanistically, EN2 might control cell proliferation and apoptosis through transcriptional regulation. EN2 is a member of the homeobox family transcription factor, and controls multiple cellular events including cellular metabolism and transmembrane transport. Therefore, EN2 may be involved in the regulation of glioma growth and survival. Interestingly, we noted that EN2 also plays a vital role in regulating cell invasion of glioma cells. Extensive infiltration of invasive cells into surrounding normal tissue is a typical feature of glioma development. Current understandings on glioma invasion reveal that the MMP family is a master regulator of tumor invasion. Particularly, MMP-9, a key member of the MMP family, is linked to metastasis in a variety of cancer types [36]. Increased expression of MMP-9 has been found in glioma tissues [37]. Thus, MMP9 is considered as an important regulatory factor for the migration and invasion of gliomas. Our work demonstrates that EN2 overexpression dramatically silences MMP-9 expression by controlling its degradation. This finding extends current knowledge to EN2 functions beyond transcriptional regulation and reveals that EN2 may also exist in the cytoplasm to control protein stability. Future work should focus on the mechanistic studies of how EN2 is involved in the protein degradation system.

## Conclusion

Our study demonstrates that EN2 is a novel suppressor of glioma tumorigenesis by decreasing cell proliferation and invasion, as well as increasing cell apoptosis. All these findings indicate that EN2 is a potential target for precision therapeutics of glioma.

## Data Availability

The datasets generated and/or analyzed during the current study are available from the corresponding author on reasonable request.
